# Case Report: Invasive Fungal Infection and Daratumumab: A Case Series and Review of Literature

**DOI:** 10.3389/fonc.2022.867301

**Published:** 2022-07-18

**Authors:** Francesca Farina, V. Ferla, S. Marktel, D. Clerici, S. Mastaglio, T. Perini, C. Oltolini, R. Greco, F. Aletti, A. Assanelli, M. T. Lupo-Stanghellini, M. Bernardi, C. Corti, F. Ciceri, M. Marcatti

**Affiliations:** ^1^ Hematology and Bone Marrow Transplantation, San Raffaele Scientific Institute, Milan, Italy; ^2^ University Vita-Salute San Raffaele, Milan, Italy; ^3^ Clinic of Infectious Diseases, Division of Immunology, Transplantation and Infectious Diseases, San Raffaele Scientific Institute, Milan, Italy

**Keywords:** fungal infection, daratumumab, multiple myeloma, mould, complications

## Abstract

Life expectancy of multiple myeloma (MM) patients has improved in last years due to the advent of anti-CD38 monoclonal antibodies in combination with immunomodulators and proteasome inhibitors. However, morbidity and mortality related to infections remain high and represent a major concern. This paper describes the “real life” risk of invasive fungal infections (IFI) in patients treated with daratumumab-based therapy and reviews the relevant literature. In a series of 75 patients we only observed three cases of fungal pneumonia. Unfortunately, the early signs and symptoms were not specific for fungal infection. Diagnostic imaging, microbiology and patient history, especially previous therapies, are critical in the decision to start antifungal treatment. Recognising the subgroup of MM patients with high risk of IFI can increase the rate of diagnosis, adequate treatment and MM-treatment recovery.

## Introduction

Infection is one of the major complications and cause of death in patients with multiple myeloma (MM) (1). This is due to immunosuppression and hypogammaglobulinemia caused by the disease itself and to treatment regimens (2). Historically, invasive fungal infections (IFIs) (3, 4) were uncommon in the course of MM treatment, however recent literature has highlighted a specific risk for this infection in the era of new therapies.

Daratumumab (Darzalex^®^, Janssen) is an IgG kappa anti CD38 monoclonal antibody approved as a monotherapy and, more importantly, in combination with immunomodulatory drugs (IMiDs) or proteasome inhibitors (PIs), both in first line and for relapsed/refractory MM (5). Daratumumab is generally well tolerated but it seems to be associated with an increased risk of infections, especially in the upper respiratory tract (6–12).

From 2017 to 2021 over 75 MM patients were treated with Daratumumab-based regimens in the Hematology and Bone Marrow Transplantation Unit at San Raffaele Institute, Milan according to approved clinical indications.

We report 3 cases of IFI; 1 probable and 2 possible infections according to the definitions from the European Organization for Research and Treatment of Cancer and the Mycoses Study Group Education and Research Consortium (EORTC/MSGERC) (3, 4):

## Case Report 1#: Probable Pulmonary Aspergillosis

A 57-years old man was admitted for progressive respiratory failure and fever.

He was diagnosed with a relapsed/refractory IgGλ MM and was undergoing the first cycle of carfilzomib-lenalidomide-dexamethasone therapy as 7^th^ line of anti MM treatment. Five years earlier he had undergone an allogenic stem cell transplant complicated during engraftment phase by fungal infection (probable pulmonary aspergillosis). Previous lines of therapy also included bortezomib, lenalidomide, pomalidomide and 9 months of daratumumab-single agent as 6^th^ line of therapy. He was treated as per cycles’ schedules with dexamethasone for more than 10 months (equivalent dose: 0.5 mg/kg/day of prednisolone). Last daratumumab infusion had occurred 40 days before admission. He was no longer receiving secondary mould-active prophylaxis and he had not graft-versus-host disease (GVHD).

Upon admission, piperacillin-tazobactam was started and, at the same time, respiratory support was provided with non-invasive ventilation. Empiric oseltamivir was added in consideration of flu epidemic period of the year. The patient was in progressive disease, with severe lymphocytopenia (0.2 x10^9^/L) but normal neutrophils count. Immunoglobulin levels were low (IgG 3.45 g/l, IgA and IgM 0.3 g/l) with normal renal function and no anemia. C-reactive protein (CRP) at admission was 2857 nmol/L (normal value < 47 nmol/L), while at the moment of IFI diagnosis CRP was 660 nmol/L.

**Figure 1:**
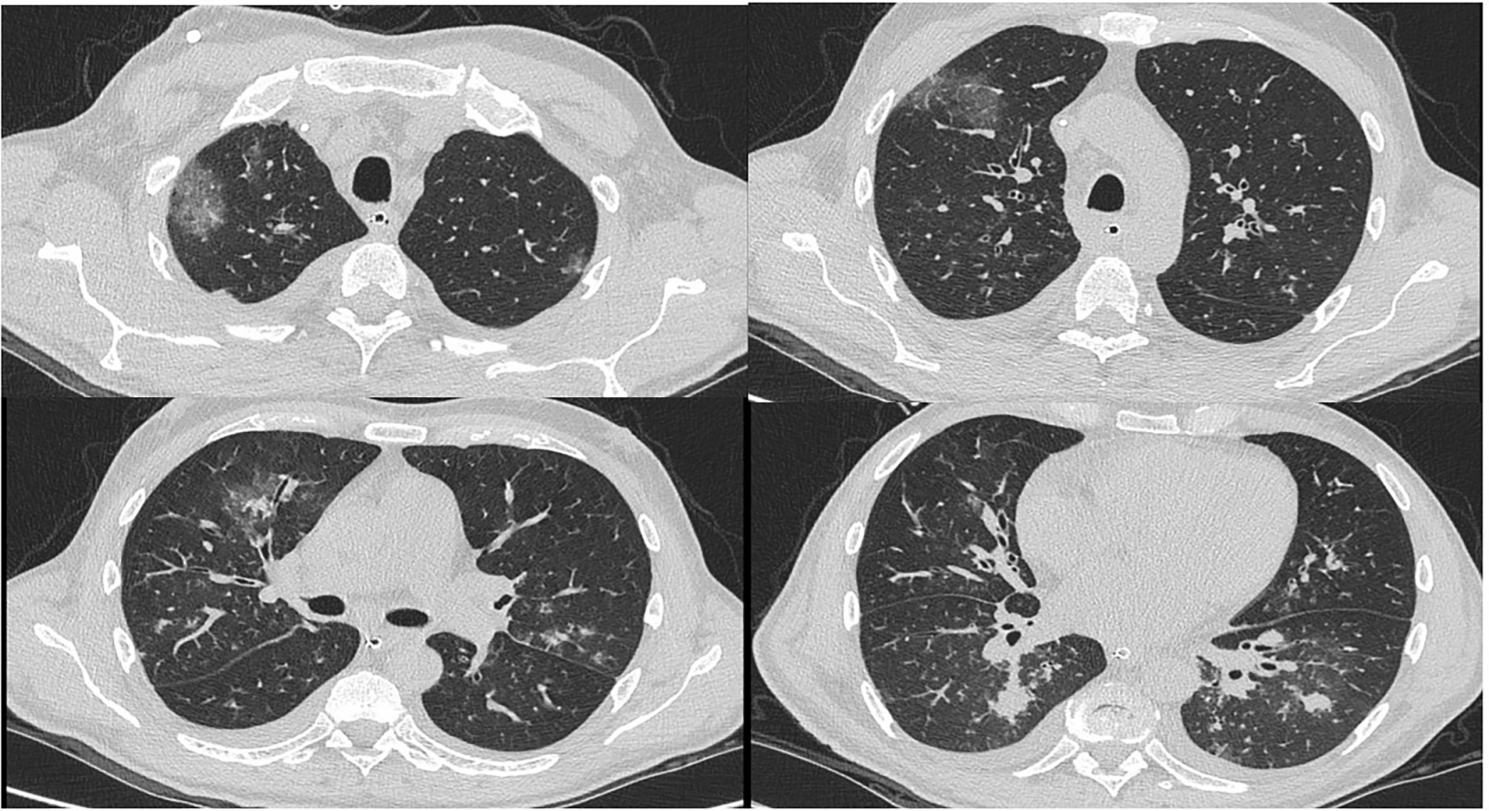
Patient 1# CT scan.

Lung computerized tomography (CT) scan showed numerous bilateral peribronchial areas with increased parenchymal density and ground glass, particularly in the left lower lobe, with also areas of parenchymal consolidation in the right base, not excavated, suggesting inflammation (**Figure 1**). We documented microbiologically Influenza A (H1N1) infection associated with pulmonary aspergillosis. Serum aspergillary antigen (AGASP) was high (1.07, normal values <0.5) and sputum culture was positive for Aspergillus Flavus. Therapy with intravenous (iv) voriconazole was started with a progressive improvement in dyspnoea, pulmonary imaging and inflammatory markers and a reduction of respiratory support requirement. AGASP levels rapidly decreased until disappearance.

Patient continued with oral voriconazole for other 60 days. At discharge lymphocytopenia was resolved with a lymphocyte count of 2.3x1^9^/L with persistent immunoparesis (IgG 3 g/l). Indeed, he received high dose of iv immunoglobulins (IVIg) as substitutive therapy during hospitalization and the following 4 months and during winter period in the next years. There was no recurrence of IFI also during subsequent lines of therapies.

## Case Report 2#: Possible Pulmonary Aspergillosis

We report the case of a 59-years old man with IgGk MM undergoing treatment with daratumumab-lenalidomide and dexamethasone as 6^th^ line of therapy (dexamethasone dose was 50% reduced after 4 months of therapy for better patient compliance). He had relapsed 7 years after allogenic stem cell transplant not complicated by GVHD and had been previously treated with both bortezomib, thalidomide and lenalidomide.

After nine months of daratumumab-based treatment, he was admitted to Haematology Department with gastroenteritis, fever and dyspnoea. Immunoglobulin levels were low (IgG 4.04 g/l, IgA and IgM 0.1 g/l) with a normal full blood count (Hb 111 g/L, neutrophils 2.7 x10^9^/L, Lymphocyte 1.1 x10^9^/L). CRP at admission was 900 nmol/L (normal value < 47 nmol/L). He had achieved more than 6 months earlier a very good partial response (VGPR) with negative imaging for bone lesions. He had received 0.35 mg/kg/day prednisolone equivalent dose for the last 150 days before IFI diagnosis. He was started empirically on antibiotic therapy consisting of iv metronidazole, azithromycin and linezolid but he experienced progressive respiratory failure and persistent fever.

**Figure 2:**
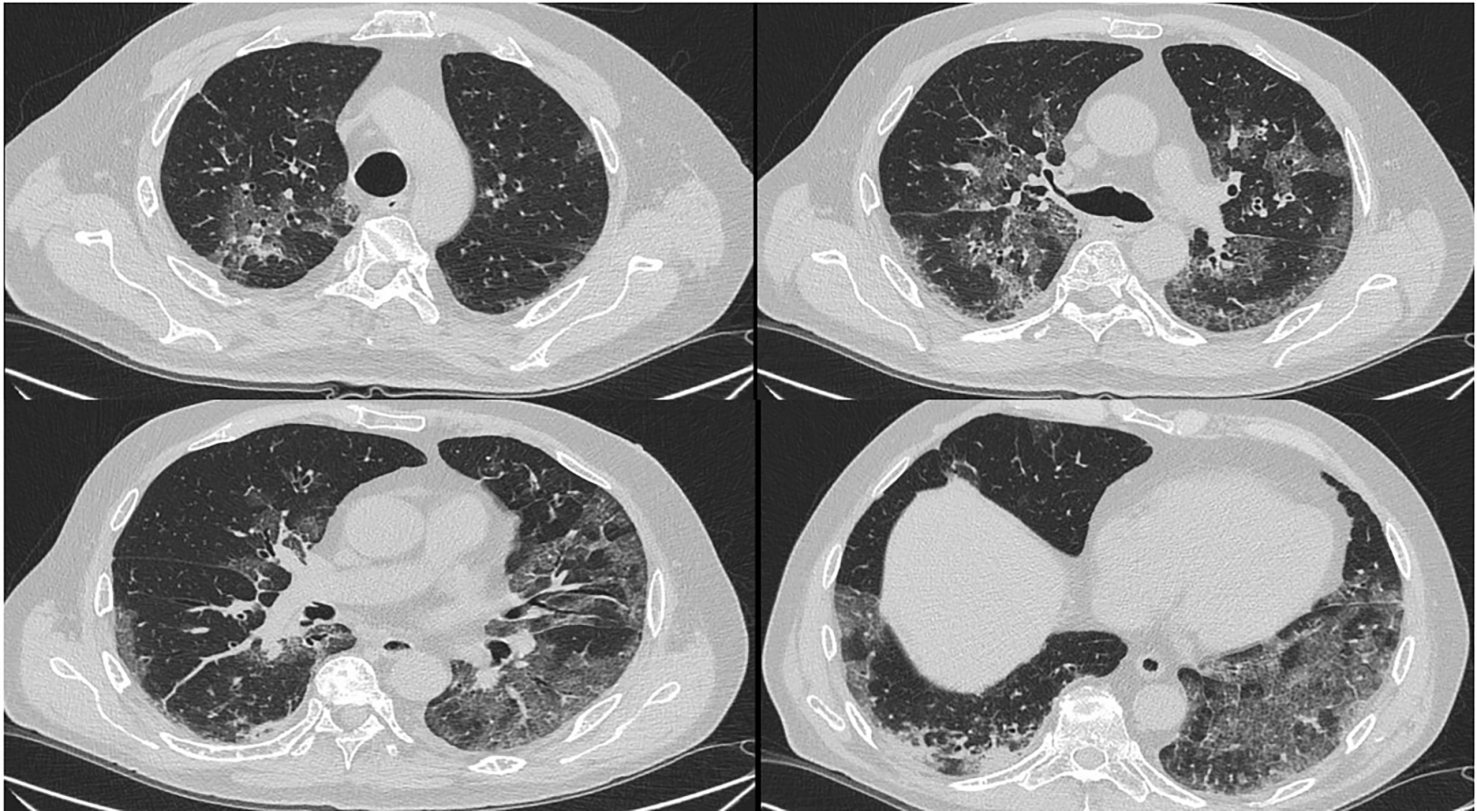
Patient 2# CT scan.

Lung CT showed small bilateral pleural effusion, areas of pulmonary thickening with air bronchogram, suggestive for inflammation, small pulmonary thickenings with peribronchial distribution, areas of increased density with a ground glass appearance and shaded micronodules (**Figure 2**). Based on these findings, iv voriconazole was started 72 hours after antibiotics. Bronchoalveolar lavage (BAL) was not performed for worsening of the clinical conditions. There was no microbiological evidence of IFI or viral reactivations and blood cultures were negative.

With antifungal therapy there was a progressive improvement in respiratory failure, cough and pyrexia: follow up CT scan showed a complete resolution of the nodules. Empiric oral voriconazole was continued for 2 months with no recurrence of signs of IFI even during further lines of MM-therapy.

## Case Report 3#: Possible Pulmonary Aspergillosis

A 75-years old man receiving daratumumab-lenalidomide and dexamethasone as first line therapy for an IgAk MM was admitted for fever, hypoxia and decrease in consciousness. Blood tests showed normal neutrophils count (3.4 x10^9^/L), lymphocytopenia (0.5 x10^9^/L) and severe hypogammaglobulinemia (1.3 g/l) that was not present at MM diagnosis. CRP at admission 647 nmol/L (normal value < 47 nmol/L). The patient had no pneumological comorbidities, respiratory function was unremarkable before starting MM-therapy and he was on antibiotics prophylaxis with levofloxacin during the first 3 cycles of therapy. He was treated with 3 cycles of MM-therapy and dexamethasone dosage was reduced at 50% according to age (0.27 mg/kg/day of prednisolone for more than 90 days) achieving a biochemical VGPR.

Meropenem and linezolid were started but 4 days later, the respiratory support was increased with non-invasive ventilation.

**Figure 3:**
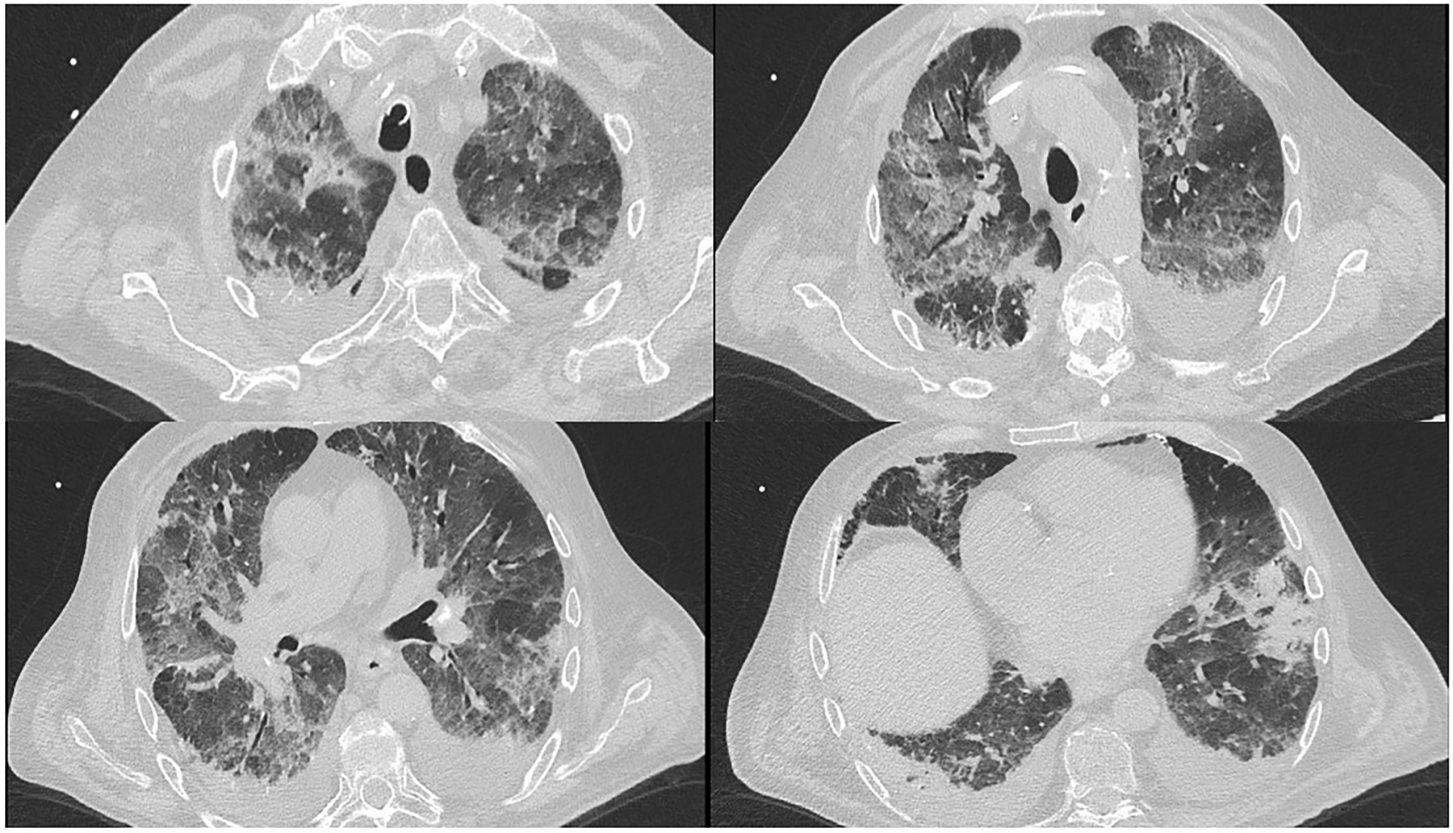
Patient 3# CT scan.

CT scan showed bilateral pleural effusion, ground glass areas and parenchymal consolidation areas. Oral posaconazole was added to broad spectrum iv antibiotics (**Figure 3**) few days before BAL obtaining therapeutic levels on blood. Serum AGASP was persistently negative.

A BAL was performed during antimicrobial therapy with no microbiologically documented infection. Very low copies of cytomegalovirus (CMV) were detectable on BAL sample but there were judged not enough for diagnosis of pulmonary CMV infection. CMV DNA on whole blood was negative and patient was not treated with antiviral therapy. Patient underwent oro-tracheal intubation due to worsening gas exchange. Broad spectrum antibiotics were continued for 10 days while empiric antifungal therapy for almost 2 months.

There was a progressive improvement, with oxygen weaning and suspension of all antimicrobial drugs. Follow up CT scan showed a regression in all ground glass and consolidation areas, lymphocyte count increased > 1.5 x10^9^/L and patient recovered and was able to resume every-day activities. After this favourable evolution, patient was able to safely re-start daratumumab-lenalidomide-dexamethasone 2 months later. He was not on secondary antifungal prophylaxis and the monitored lung CT scan at 4 months from the IFI was negative. Until now, after other 5 cycles of MM-therapy there was no IFI outbreak, patient continued substitutive IVIg according to blood dosage (median IgG 3.2 g/l).

## Discussion

Infections are among the major causes of morbidity and mortality in MM patients (13).

Susceptibility to infections in MM is multifactorial, including hypogammaglobulinemia and aberration of dendritic cell function, B and T cell immunity (14, 15). The importance and interconnected responses of innate and adaptive immune system in IFI- protection are being widely investigated (15). Patient characteristics such as old age, multiple comorbidities, state of the disease and immunosuppressive treatments, confer major risk and increase the severity of infections (14).

In the past years, the incidence of fungal infections (especially aspergillosis) was reported as significant in MM treated with conventional chemotherapy, with a mortality of 50% (16). Another study described 98 cases of neutropenia related-invasive aspergillosis (IA) in MM patients after chemotherapy or autotransplant, with a 63.4% response to antifungal therapy (17).

From the advent of biological therapies, IFI epidemiology has changed.

A retrospective study on lymphoproliferative diseases showed a rate of 5.6% IFI in 248 MM patients; all IFI were IA (18): first line with PIs and further lines of therapy with IMIDs were equally distributed (8 cases vs 6 cases).

The SEIFEM2004 study evaluated the incidence and outcome of IFI in haematological malignancies in Italy. 1616 myeloma patients were included with 7 patients diagnosed with IFI (0.5%, of whom 4 were mould infection) (19). Notably, the incidence of IFI after allogeneic stem cell transplant (SCT) can be as high as 20%, with a mortality rate of 50- 80%, compared to 2-6% in the autologous SCT setting (2-6%) (20). The French SAIF network identified 5 MM patients who received allo-SCT with IA on a total population of 424 allotransplants (21).

Indeed, IA often occur as a result of cumulative immunosuppression, neutropenia and prolonged use of steroids that are part of all therapeutic regiments for MM (often 40 mg or 20 mg of dexamethasone per week). Clinical manifestation are reported to be atypical with micronodules, ground-glass opacities and tree-in-bud infiltrates (22).

A single-centre study published in 2015 studied IFI in MM patients treated with novel agents (IMiDs and PIs): the rate of invasive mould infection and IA were 0.8% and 0.3% respectively. IFI rates were reported to be 2.2-2.5% in relation to the use of auto transplant as consolidation with a mortality of 44%. Multivariate analysis showed that the only risk factor for IFI was having received more than 3 lines of therapy with a rate of IFI of 15% in this setting. The authors noted that, despite the lack of administration of mould-active prophylaxis, the rate of IA and mould infections were low and IFI occurred mostly during disease progression and in patients with a median of 5 lines of therapy (23).

Another recent single-centre study reported a 3.5% incidence of proven or probable IFI in MM patients with high early mortality. Patients were treated with both PIs, IMIDS, conventional chemotherapy and auto or allo-SCT. Of the 22 IFI reported, 31.8% were mould infections, and among these 71% were pulmonary IA. Multivariate analysis showed that light chain disease, low haemoglobin level, low serum albumin and previous allogenic stem cell transplant were associated with IFI (24).

Randomized clinical trials (RCT) on daratumumab in relapsed/refractory MM documented an incidence of grade 3 and 4 infections of 21.4% and 28.3% respectively with a rate of grade 3-4 pneumonia of 9% (6, 7). An analysis on RCT with daratumumab in first line demonstrated that the risk of infection is increased especially in patients with age >= 75 years, elevated baseline alanine aminotransferase, high LDH and low albumin levels (25).

Moreover, in addition to previously reported risk factors, treatment with Daratumumab reduced both Natural Killer (NK) cells and other CD38-expressing immune cells and cytotoxic T lymphocytes (26–28), providing a biological explanation of a possible increased risk of infection in this population especially for viral infection. Some evidences also reported that NK cells play an important role in the antifungal host response with direct fungal damage and the release of multiple cytokines that activate the immune system (29). Despite the role of Daratumumab-impaired NK and T cells response in fungal infections is not fully demonstrated, it is possible that it may play a role in increasing the risk of IFI in patients, although this risk cannot be separated from the concomitant use of steroids and other biological therapies.

A retrospective study evaluated the incidence of infections in patients treated with daratumumab-containing regimens: rate increased from 26% to 56% when daratumumab was used as single agent or combined with other agents (30); no data on IFIs were availed. Another “real word” study on rates of infections and severe lymphopenia in 100 patients treated with daratumumab showed only 1 patient with IFI (fungal meningitis) (31). The authors showed a higher rate of severe lymphopenia in daratumumab-based regimens combined with IMIDs, with higher rates of serious infection in this patient population.

A recent study also evaluated the role of hypogammaglobulinemia in daratumumab-based therapies, both in relapsed/refractory MM patients and during first line treatment (respectively 88% and 12%). Daratumumab causes a rapid decrease in uninvolved free light chain and immunoglobulin levels with a nadir within 2-4 months. The authors demonstrate that decreased poly-IgG levels after treatments and high risk cytogenetics were associated with higher risk of infections in multivariate analysis. Infections were mainly respiratory and often self-resolving. There was a 1.2% incidence of invasive fungal infections (1 patient treated with Daratumumab monotherapy and 1 with Daratumumab+PIs) (32).

Some evidence showed that administration of intravenous immunoglobulins at substitutive doses can reduce infection rate (33), but the impact of this practice is not clearly established, especially for patients in first line of treatment.

Due to the low rate of IFI in patients with MM, there is currently no consensus on the role of antifungal prophylaxis, especially mould active (1, 34, 35). The study by Teh et al. (23), showing a 15% risk of developing an IFI after 3 or more lines of treatments, suggests the opportunity to consider surveillance and antifungal prophylaxis in high-risk patients. On the other hand, patients who receive high-dose chemotherapy and develop severe mucositis could require yeast prophylaxis (35).

We presented 1 case of probable pulmonary aspergillosis and 2 cases of possible pulmonary fungal infection. According to EORTC/MSGERC consensus (4), our patients had more than 2 host factors for IFI that are well recognized not only as risk factors but also as a clear predisposition to IFI. All patients received a significant dose of steroids for more than 60 days. Two patients were heavily pre-treated and underwent previous allogenic stem cell transplantation. Two patients were receiving immunoglobulins replacement therapy due to the low levels possibly related to daratumumab therapy or MM itself. All patients had from moderate to severe lymphocytopenia that was resolved during the IFI episode.

In 4 years’ time, we have only observed 3 cases of IFI over 75 patients treated with Daratumumab for MM. Two of them previously received allo-transplant. The incidence is 0.04% until now, however we need to extend the observation period to evaluate the impact of daratumumab in first line of treatment and in patients treated continuously for more than 3 years of therapy and in whole MM population.

Based on our observation and on published data, even if there is a biological rational, it is impossible, at the moment, to establish whether patients treated with daratumumab have a higher risk of IFI or the risk is a sum of the variety of host factors that MM-patients usually accumulate. The evaluation of IFI risk is complex also due to the fact that patients receive various treatment classes in combination even in previous line of therapy, thus adding up the specific infection risk for each class.

The possible role of IVIg infusions and lymphocyte to neutrophils ratio in prevention of infections, especially IFI, in MM patients needs to be extensively studied. Published data and our single centre experience confirm at the moment that primary mould-active prophylaxis is not recommended in all MM population. Role of antifungal prophylaxis need to be established case by case looking at all host factors.

Nevertheless, the cases presented underline the importance of early recognition of signs and symptoms of IFI especially in MM patients at high risk suggesting an active serum AGASP surveillance in relapsed/refractory MM and persistent severe lymphopenia and a role of early lung CT scan in persistent fever without other microbiological explanation.

Further studies are necessary to better recognize the epidemiology of IFI in this setting and clearly recognize a sub-population at higher risk.

## Conclusions

IFI in MM patients is a rare complication that historically occurred in highly pre-treated patients with important immunosuppression related to therapies and progressive disease. There is no actual evidence of a direct increased risk of IFI in Daratumumab-treated patients: at the moment, the majority of patients are affected by relapsed/refractory MM in which we cannot split the effect of previous and concomitant cytotoxic or biological therapies, concurrent neutropenia and hypogammaglobulinemia, autologous or allogenic HSCT and the role of the underlying disease. Currently, there is no strong evidence of which MM population can benefit from an antifungal prophylaxis, especially mould-active. A risk-adapted selection of population, taking in consideration tumour and host risk factors, will help the clinician in the management of suspected IFI. Prophylactic immunoglobulin infusion is currently suggested to reduce the risk of infections in patients with hypogammaglobulinemia. However, the progressive increase in use of daratumumab-based therapies adding a novel IFI risk factor will possibly change the epidemiology of fungal infections and result in a different antimicrobial approach. Further “real life” observations are necessary to understand the impact of anti CD38 therapy and better recognize patient at risk.

## Data Availability Statement

The raw data supporting the conclusions of this article will be made available by the authors, without undue reservation.

## Ethics Statement

Written informed consent was obtained from the individual(s) for the publication of any potentially identifiable images or data included in this article.

## Author Contributions

FF collected the data and wrote the manuscript. All authors have approved the final version of the manuscript and contributed to patients’ clinical care.

## Conflict of Interest

The authors declare that the research was conducted in the absence of any commercial or financial relationships that could be construed as a potential conflict of interest.

## Publisher’s Note

All claims expressed in this article are solely those of the authors and do not necessarily represent those of their affiliated organizations, or those of the publisher, the editors and the reviewers. Any product that may be evaluated in this article, or claim that may be made by its manufacturer, is not guaranteed or endorsed by the publisher.
